# The compositions, characteristics, health benefits and applications of anthocyanins in *Brassica* crops

**DOI:** 10.3389/fpls.2025.1544099

**Published:** 2025-02-17

**Authors:** Xinjie Li, Fan Wang, Na Ta, Jinyong Huang

**Affiliations:** ^1^ School of Life Sciences, Zhengzhou University, Zhengzhou, China; ^2^ School of Modern Agriculture and Biotechnology, Ankang University, Ankang, China

**Keywords:** *Brassica* crops, anthocyanin, acylation, natural colorants, health benefit

## Abstract

*Brassica* crops, well known for their nutritional and medicinal value, encompass a diverse range of species and varieties, many of which are rich in anthocyanins. These flavonoid pigments not only contribute to the vibrant colors of *Brassica* plants but also possess significant antioxidant, anti-inflammatory, and neuroprotective properties. This review provides an in-depth analysis of the distribution, composition, and health benefits of anthocyanins in *Brassica* crops, highlighting their potential applications in the food industry and medicine. We discuss the accumulation patterns of anthocyanins in various *Brassica* tissues, the influence of genetic and environmental factors on their concentration, and the impact of acylation on their stability and biological activities. This review also explores the antioxidant capacity and cardioprotective effects of *Brassica* anthocyanins, as well as their roles in protecting against hepatic and renal injury and promoting neuroprotection. Furthermore, we examine the use of anthocyanins as natural food colorants and their integration into intelligent packaging for the real-time monitoring of food freshness. Our findings underscore the multifaceted benefits of *Brassica* anthocyanins, positioning them as key components in the development of functional foods and sustainable food systems.

## 1 Introduction

*Brassica* is an important genus of the *Brassicaceae* family that has been used as a food source and medicinal compound in Eurasia since ancient times (Cheng et al., 2014). *Brassica* plants are currently used in vegetable and oil production in more than 150 countries; provide edible roots, leaves, stems, buds, flowers, and seed products; and play important roles in agricultural production (Velasco et al., 2017; Rakow, 2004).

The genus *Brassica* consists of six widely cultivated species: *Brassica rapa* L., *Brassica oleracea* L., *Brassica napus* L., *Brassica nigra*(L.) K. Koch, *Brassica carinata* A. Braun, and *Brassica juncea*(L.) Czern. These species are utilized globally as vegetables, oilseed crops, condiments and fodder. *B. napus*, *B. carinata* and *B. juncea* are allotetraploid species that evolved from the natural hybridization of three diploid progenitors (*B. oleracea*, *B. rapa*, and *B. nigra*). The versatile *B. oleracea*, a cornerstone of vegetable cultivation, encompasses an array of distinct varieties, notably broccoli (var. *italica*), cauliflower (var. *botrytis*), kale (var. *acephala*), cabbage (var. *capitata*), kohlrabi (var. *gongylodes*), and Brussels sprouts (var. *gemmifera*) (Šamec et al., 2016). *B. rapa* encompasses diverse forms of vegetables, including the versatile turnip, the highly popular Chinese cabbage, and the delicate pak choi, in addition to varieties utilized as forage crops and for oilseed production (Escribano-Bailón et al., 2004). This species displays remarkable versatility, accommodating various culinary and agricultural purposes. They are not only delicious but also rich in nutrients and constitute an important part of people’s daily diet (Nagaharu, 1935). Some *Brassica* seeds can be used to extract oil, such as rapeseed, which produces high-quality oil with a variety of nutritional benefits. In addition, *B. carinata* and *B. juncea* are two important *Brassica* crops. The *B. carinata* seed, a unique nonedible oil crop, is cultivated primarily for the production of oil-derived products, notably in the synthesis of biodiesel (Mekuriaw and Abera, 2024). Notably, *B. carinata* has traditional medicinal value and is well known for its therapeutic potential in addressing wounds and alleviating gastrointestinal disturbances (Nakakaawa et al., 2023). The vitamin A content in *B. juncea* is important for maintaining good vision and contributes to healthy skin. Furthermore, the dietary fiber in *B. juncea* helps promote intestinal health, prevent constipation, help lower serum cholesterol levels, and reduce the risk of cardiovascular disease.

In addition to agriculture, studies have shown that *Brassica* crops have a wide range of applications in the food industry and medicine. For example, *B. campestris* L., *B. juncea*(L.) Czern., *B. campestris* var. *purpuraria*, and *B. oleracea* var. *italica* have medicinal value, and *B. alboglabra* L. H. Bailey and *B. rapa* ssp. *pekinensis* can be used to treat certain diseases or as health foods (Kapusta-Duch et al., 2012; Zhao A. et al., 2022; Zhao Y. et al., 2022; Mahn and Reyes, 2012). *Brassica* species are well known for their abundant accumulation of essential nutrients and bioactive phytochemicals, encompassing a diverse array of vitamins, minerals, and compounds such as indole phytoalexins, phenolic acids, and glucosinolates (Salehi et al., 2021). These constituents confer a multitude of biological benefits to *Brassica* plants, particularly in safeguarding cardiovascular health and mitigating cancer risk. The antimicrobial properties of these compounds (Vale et al., 2015), coupled with their potent antioxidant (Bhandari and Kwak, 2015; Soengas et al., 2012) and anticancer activities (Hafidh et al., 2013; Thangam et al., 2013), underscore their importance in promoting overall wellness. Extensive epidemiological investigations involving human subjects have revealed a compelling inverse correlation between increased consumption of *Brassica* vegetables and the incidence of cancer, specifically highlighting their chemoprotective potential against malignancies of the lung, stomach, colon, and rectum (Avato and Argentieri, 2015).

A variety of natural pigments exist in nature, including chlorophylls, carotenoids, flavonoids, and quinone derivatives, which provide rich colors and play important physiological roles in living organisms (Honda and Moriya, 2018). Owing to their unique natural properties and healthy and harmless characteristics, natural plant pigments have gradually replaced some chemically synthesized pigments and have become important coloring agents in modern industry. Anthocyanins are a class of flavonoid pigments that are widely found in plants and give them vibrant colors ranging from blue to purple. The basic structure of anthocyanins consists of two benzene rings connected by a pyran ring with multiple substituents, and their color-forming principle is affected by a variety of factors, such as pH, cochromatism, and molecular modification (Francis F. J., 1989; Roy and Rhim, 2021; Lv et al., 2022), which work together to make anthocyanins colorful in plants. Anthocyanins have a variety of biological activities, such as antioxidant, anti-inflammatory, and free radical scavenging and antiaging activities (Zhang et al., 2020), which has positive implications for the prevention and treatment of certain diseases (Escribano-Bailón et al., 2004). In addition, it has also been shown that anthocyanins can promote the regeneration of retinal cells, prevent myopia, improve vision, and have multiple benefits for human health (Alappat and Alappat, 2020).

In *Brassica* crops, a very large number of varieties are characterized by the accumulation of rich anthocyanins. These varieties usually have a bright red–blue–purple color and are highly ornamental, nutritional, and medicinal value (Favela-Gonzalez et al., 2020). Considering the universality of anthocyanin accumulation in the *Brassica* genus and the biological value of anthocyanins, this review aims to provide an overview for a better understanding of the organizational distribution, composition, and health benefits of anthocyanins in *Brassica* crops and their potential applications in the food industry and medicine.

## 2 Anthocyanin accumulation in *Brassica* crops

**Figure 1:**
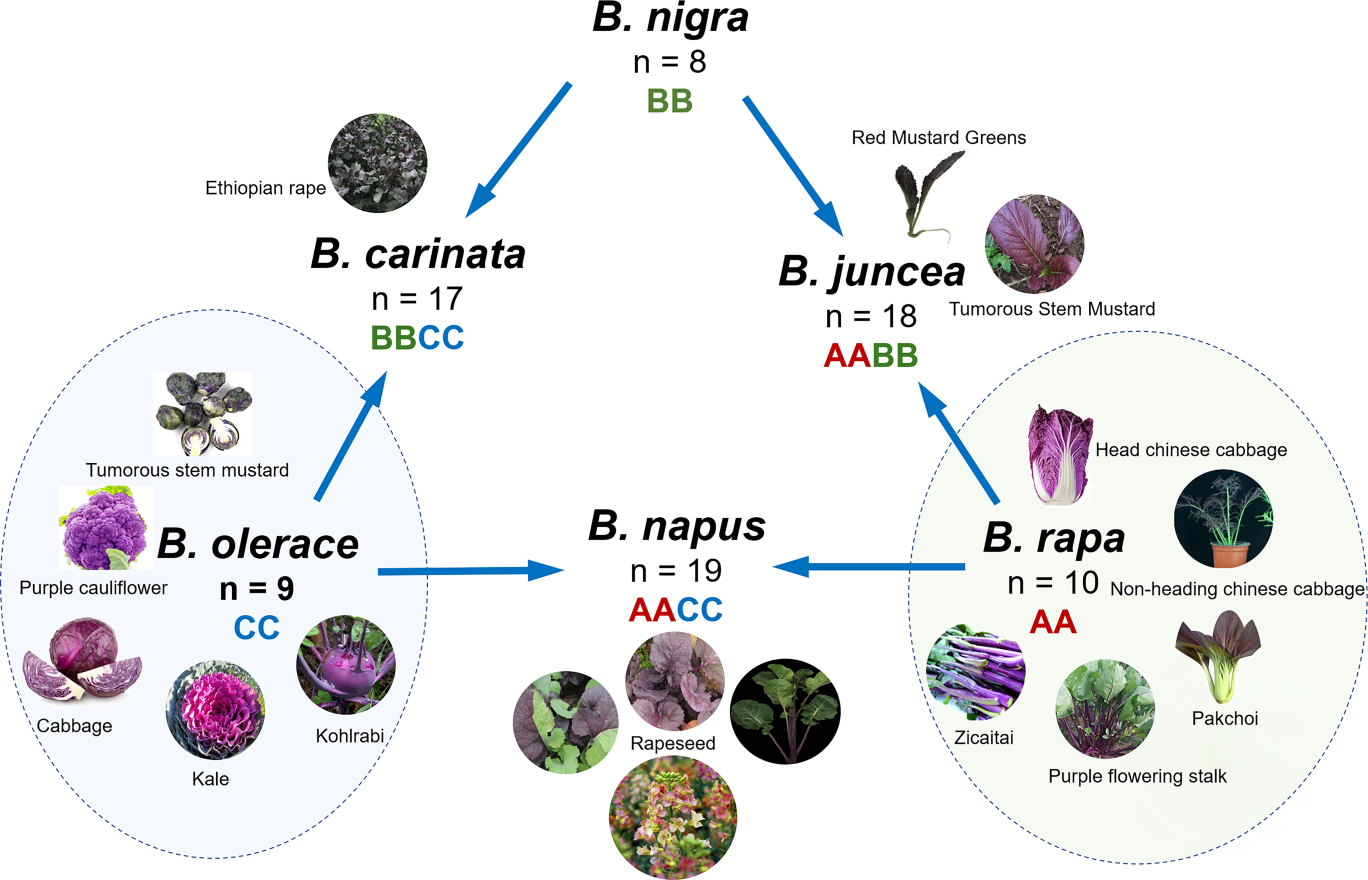
Purple varieties/cultivars in *Brassica* crops.

Anthocyanins accumulate in tissues and parts that often appear red or purple and are the main pigments responsible for the coloration of plant petals, leaves, stems, and fruits (Escribano-Bailón et al., 2004). In species of the *Brassica* genus, numerous varieties with anthocyanin accumulation have been discovered (**Table 1**; **Figure 1**). *B. oleracea*, a major vegetable species, includes the most purple or red varieties/cultivars, the most studied of which are red cabbage (var. *capitata*) (Song et al., 2018; Ghareaghajlou et al., 2021), purple broccoli (Liu et al., 2020; Rahim et al., 2019) and purple cauliflower (Chiu and Li, 2012), purple kale (var. *acephala*) (Zhang et al., 2012), purple kohlrabi, and purple Brussels sprouts (var. *gemmifera*), which have anthocyanin accumulation. For *B. rapa*, a diverse array of purple-hued cultivars showcase remarkable anthocyanin pigmentation, including the purple flowering stalk (*Campestris* var. *purpurea Bailey*) (Guo et al., 2023), purple head Chinese cabbage (He Q. et al., 2020), *B. rapa* L. *ssp. chinensis* (He Q. et al., 2020), nonheading Chinese cabbage (ssp. *Chinensis Makino* var. *mutliceps Hort.*) (Zhao Y. et al., 2022), *B. rapa* ssp. Parachinensis (He et al., 2016), *B. rapa subsp. chinensis* (Jeon et al., 2018), bok choy (var. *chinensis*) (Zhang et al., 2014), and *B. rapa* L. ssp. *chinensis* var. purpurea (Guo et al., 2015). In addition, purple tumorous stem mustard (var. tumida Tsen et Lee) (Xie et al., 2014) and red mustard greens (Coss variety) (Lin et al., 2011) have been reported for *B. juncea*, and mustard with only purple leaf veins and leaf edge cracks has also been studied and reported in *B. juncea*(Zhang et al., 2022). *B. napus* has several anthocyanin-enriched variants, including purple-leaved *B. napus*(Huang et al., 2024) and purple-stemmed *B. napus*(Chen et al., 2022), and variants with diversely colored petals resulting from anthocyanin accumulation have been developed for ornamental purposes (Yin et al., 2019; Zeng et al., 2023; Cui et al., 2024). Additionally, *B. carinata* with purple leaves has gradually gained attention because of its ornamental and nutritional value (Mushtaq et al., 2016).

**Table 1:** Anthocyanin concentration in *Brassica* crops.

Scientific name	Common name	Cultivars or lines	Anthocyanin-enriched Tissue	Concentration^a^(g/Kg)	Detection method^b^	Reference
*Brassica rapa* L.	Head Chinese cabbage	11S91	Seedling midrib	0.042 FW	pH differential (Cy3G)	(He Q. et al., 2020)
*Brassica rapa* var. *chinensis*	Purple bok choy	Zi He	Sprout	3.130 DW	HPLC (Cy3,5diG)	(Zhang et al., 2014)
*Brassica rapa* ssp. *chinensis L.*	Pak choi	ZBC	Leaves	0.126 FW	Colorimetric (Cy3G chloride)	(Tan et al., 2023)
		RSH	Leaves	0.480 FW	Colorimetric (Cy3G chloride)	(Song et al., 2020)
		PHXW	Leaves	0.420 FW	Colorimetric (Cy3G chloride)	(Song et al., 2020)
		RWTC	Leaves	0.390 FW	Colorimetric (Cy3G chloride)	(Song et al., 2020)
		PQC	Leaves	0.290 FW	Colorimetric (Cy3G chloride)	(Song et al., 2020)
*Brassica rapa* L. ssp. *chinensis Makino* var. *mutliceps Hort.*	Non-heading Chinese cabbage		Leaves	19.400 FW	pH differential (Cy3G chloride)	(Zhao Y. et al., 2022)
*Brassica rapa* L. ssp. *pekinensis*	Chinese cabbage	85772	Leaves	4.380 DW	pH differential (Cy3G equivalent)	(Yeo et al., 2023)
*Brassica rapa* L. ssp. *pekinensis*	Reddish purple Chinese cabbage	RPCC	Outermost leaf	10.17 DW	HPLC (Cy3G)	(Rameneni et al., 2020)
		RPCC	Innermost leaf	32.310 DW	HPLC (Cy3G)	(Rameneni et al., 2020)
*Brassica rapa* L. ssp. *pekinensis*	Chinese cabbage	11S91	Leaves	0.018-0.394 FW^c^	pH differential (Cy3G)	(He et al., 2016)
			Innermost leaves	0.394 FW	pH differential (Cy3G)	(He et al., 2016)
*Brassica campestris* L. var. *purpurea Bailey*	Purple flowering stalk		Stalk peel	1.563 DW	HPLC (Cy3G chloride)	(Guo et al., 2023)
*Brassica campestris* L. ssp. *Chinensis* var. utilis Tsen et Lee	Flowering Chinese cabbage	95T2	Seedling midrib	0.158 FW	pH differential (Cy3G)	(He Q. et al., 2020)
			Leaves and stems	0.634 FW	pH differential (Cy3G)	(He et al., 2016)
*Brassica campestris* ssp*. chinensis*	Non-heading Chinese cabbage		Leaves	0.076 FW	pH differential (Cy3G)	(Tang et al., 2022)
*Brassica campestris (*syn. *Brassica rapa)* ssp. *chinensis*	Non-heading Chinese cabbage	ZBC	Leaves	0.320 FW	pH differential (Cy3G)	(Zhou et al., 2024)
*Brassica oleracea* L. var. *acephala*	Ornamental kale	Y007-P-24	Leaves	0.126 DW	pH differential (Cy3G)	(Zou et al., 2023)
*Brassica oleracea* L. var. *italica*	Broccoli	Viola	Sprout	0.006 FW	HPLC (Cy3G)	(Moreno et al., 2010)
		Marathon	Sprout	0.003 FW	HPLC (Cy3G)	(Moreno et al., 2010)
		Nubia	Sprout	0.003 FW	HPLC (Cy3G)	(Moreno et al., 2010)
		Intersemillas	Sprout	0.002 FW	HPLC (Cy3G)	(Moreno et al., 2010)
		Plenck	Sprout	0.127 DW	pH differential (Cy3G)	(De La Fuente et al., 2019)
*Brassica oleracea* L.var. *botrytis*	Violet cauliflower	Grafitti	Head of cauliflower	0.739 FW	pH differential (Cy3G)	(Volden et al., 2009)
		Violetto di Catania	Head of cauliflower	0.077 FW	pH differential (Cy3G)	(Scalzo et al., 2008)
		Natalino	Head of cauliflower	0.018 FW	pH differential (Cy3G)	(Scalzo et al., 2008)
		Sammartinaro	Head of cauliflower	0.040 FW	pH differential (Cy3G)	(Scalzo et al., 2008)
*Brassica oleracea* var. *gongylodes*	Purple kohlrabi	Early purple Vienna	Peel	0.786 DW	HPLC (Cy3G)	(Park et al., 2012)
		Azur- Star	Peel	0.030 DW	HPLC (Cy3G)	(Rahim et al., 2018)
		Kolibri	Peel	3.020 DW	HPLC (cyanidin 3,5-diglucoside)	(Zhang et al., 2015)
*Brassica oleracea* var.*acephala f. tricolor*	Purple kale	Red Dove	Leaves	1.730 FW	pH differential (Cy3G)	(Zhang et al., 2015)
*Brassica oleracea* var. *sabellica* L.	Green curly kale		Sprout	0.014 DW	pH differential (Cy3G)	(De La Fuente et al., 2019)
*Brassica oleracea* L. var. *capitata* L. *f. rubra*	Red cabbage	Langedijker Polona	Leaves	2.260 and 6.290 DW^d^	HPLC (Cy3G)	(Wiczkowski et al., 2016)
		Kissendrup	Leaves	1.810 and 3.730 DW^d^	HPLC (Cy3G)	(Wiczkowski et al., 2016)
		Koda	Leaves	1.540 and 3.320 DW^d^	HPLC (Cy3G)	(Wiczkowski et al., 2016)
		Kalibos	Leaves	1.130 and 3.170 DW^d^	HPLC (Cy3G)	(Wiczkowski et al., 2016)
		Langedijker Dauer 2	Leaves	2.140 and 4.240 DW^d^	HPLC (Cy3G)	(Wiczkowski et al., 2016)
		Violetto di Catania	Leaves	0.756 DW	pH differential (Cy3G)	(Scalzo et al., 2008)
		Langedijker	Leaves	2.320 DW	HPLC (Cy3G)	(Wiczkowski et al., 2013)
		Cardinal	Leaves	1.820 FW	pH differential (Cy3G chloride)	(Yuan et al., 2009)
		Primero	Cabbage heads	11.110/10.260 DW, 1.090/1.040 FW^e^	pH differential (Cy3G)	(Tang et al., 2022)
		Integro	Cabbage heads	16.600/16.370 DW, 1.850/1.880 FW^e^	pH differential (Cy3G)	(Ahmadiani et al., 2014)
		Azurro	Cabbage heads	13.920/12.170 DW, 1.440/1.370 FW^e^	pH differential (Cy3G)	(Ahmadiani et al., 2014)
		Kosaro	Cabbage heads	12.470/10.010 DW, 1.280/1.150 FW^e^	pH differential (Cy3G)	(Ahmadiani et al., 2014)
		Cairo	Cabbage heads	13.890/12.560 DW, 1.530/1.680 FW^e^	pH differential (Cy3G)	(Ahmadiani et al., 2014)
		Bandolero	Cabbage heads	15.170/15.120 DW, 1.650/1.650 FW^e^	pH differential (Cy3G)	(Ahmadiani et al., 2014)
		Buscaro	Cabbage heads	17.800/12.360 DW, 1.700/1.370 FW^e^	pH differential (Cy3G)	(Ahmadiani et al., 2014)
*Brassica juncea*(L.) *Czern.*	Red mustard		Sprout	0.364 DW	pH differential (Cy3G)	(De La Fuente et al., 2019)
*Brassica juncea* var. tumida Tsen et Lee	Purple mustard	Zi Ying	Sprout	1.929 FW	pH differential (Cy3G)	(Xie et al., 2014)
*Brassica juncea*		ZY	Leaves	0.744 FW	Colorimetric (Cy3G chloride)	(Heng et al., 2020)
*Brassica napus* L.	Rapeseed	p1029	Leaves	0.633 FW	Colorimetric (Cy3G chloride)	(Heng et al., 2020)
		PR01	Leaves, stem	12.430 DW, 1.380 DW	HPLC (Cy3G)	(Fu et al., 2022)

a The abbreviations DW and FW following the numbers indicate whether the measurements were taken on a dry weight (DW) or fresh weight (FW) basis of the material.

b The brackets indicate the standard used for anthocyanin quantification. Cy3G, cyanidin-3-glucoside; Cy3,5diG, cyanidin 3,5-diglucoside.

c Anthocyanin concentrations differ from those of internal heading leaves to external heading leaves.

d Anthocyanin concentration in different growing seasons.

e Anthocyanin concentration at two different harvest times (13 and 21 weeks).

In these purple *Brassica* crops, anthocyanins accumulate mostly in nutrient-containing organs, i.e., leaves and stems (**Figure 1**). In the case of leaves, the deposition forms of anthocyanins are diverse, and their presence results in red, purple, blue, pink, and other colors (Ren and Zemel, 2015). Ornamental kale, a horticultural variety of *B. oleracea*, has leaves in a range of colors, such as white, purple, pink, yellow, and red, leading to high ornamental value. Red cabbage is well known for its bright purple leaves, and its bright color and high nutritional value make it a common healthy ingredient in salads (Hanschen, 2020). Purple ornamental cabbage (*B. oleracea* var. *acephala*) is characterized by green outer leaves and purple inner leaves, which are attributed to the specific accumulation of anthocyanins in the inner leaves (Jin et al., 2019). Moreover, purple kale has dark purple leaf veins resulting from the special accumulation of anthocyanins (Barcena et al., 2019). Similarly, in the anthocyanin-enriched varieties, the entire aboveground parts of the flowering stalk, especially the stems, exhibit a vibrant purple color, making them widely favored by consumers. Bok choy (Zhang et al., 2014), non-heading Chinese cabbage (Zhao Y. et al., 2022), and red flat cabbage (*B. rapa* L. subsp. *narinosa*) (Park et al., 2021) primarily accumulate anthocyanins on the surface of their leaf blades, which mix with chlorophyll to produce a dark purple hue. The accumulation of anthocyanins in the stem bark results in purple stems, which is also a relatively common phenomenon in *Brassica* crops. In addition to purple-stalked Chinese kale (*B. oleracea* var. *alboglabra*), which displays a rich purple color only in its stem (Tang et al., 2024), rapeseed with purple flower stalks resulting from the specific accumulation of anthocyanins has increased its value as a vegetable (Chen D. et al., 2023). In particular, purple kohlrabi shows abundant anthocyanin accumulation in the epidermis of swollen stems (Zhang et al., 2015). In the two cabbage varieties, broccoli and cauliflower, anthocyanin accumulation was observed only in the flower buds of certain cultivars. To date, colored petals caused by anthocyanin accumulation have been reported only in rapeseed (Cui et al., 2024; Zeng et al., 2023; Yin et al., 2019). In general, anthocyanins most commonly accumulate in the epidermal layers of various aboveground organs in *Brassica* crops, including the upper and lower sides of the leaves, stems, swollen stems, and buds (Chiu and Li, 2012), which is due to the exposure of the epidermis of these tissues to light.

The concentrations of anthocyanins reported in *Brassica* crops vary greatly among varieties, and the levels of these anthocyanins are summarized herein (**Table 1**). Ahmadiani et al. (2014) reported that the anthocyanin content of seven red cabbage cultivars ranged from 11.110 to 17.800 g/kg dry weight (DM), and these values for fresh matter were approximately 1.090 and 1.700 g/kg fresh weight (FM), respectively, and did not increase with time. The total anthocyanin content was found to be 0.739 g/kg for the head of cauliflower in Violet cauliflower and 3.02 g/kg dry weight for the swollen stem peel in Kolibri (Park et al., 2012; Zhang et al., 2015). Although anthocyanins are absent in adults, the sprouts of broccoli and kale are rich in anthocyanins (0.127 and 0.014 g/kg DW, respectively) (De La Fuente et al., 2019). The anthocyanin accumulation in the purple stem bark of purple flowering stalks, highly valued by consumers for its vibrant color, reaches 1.563 g/kg Dw (Guo et al., 2023).

The levels of anthocyanin accumulation vary across different plant growth stages and are influenced by genetic factors, environmental conditions, physiological stress responses, and the nutritional state of the plant (Kim et al., 2021; Chalker-Scott, 2008). The anthocyanin content in *Brassica* crops has been shown to fluctuate depending on the cultivar (Moreno et al., 2010), specific plant organ (Park et al., 2012; Rahim et al., 2018), season of growth (Guarise et al., 2019; Wiczkowski et al., 2014), and environmental growth conditions (Baenas et al., 2015), even within the same species. The most common phenomenon of anthocyanin accumulation is in the organ epidermis (Guo et al., 2023; Park et al., 2012; Zhang et al., 2015; Rahim et al., 2018; Fu et al., 2022), possibly due to the regulation of anthocyanin accumulation by light. In the red cabbage variety ‘Langedijer Polona’, the anthocyanin content measured in 2009 (6.290 g/kg DW) was threefold greater than that measured in 2008 (2.260 g/kg DW) (Wiczkowski et al., 2014), likely due to differences in cultivar characteristics and growing conditions. The outermost and innermost leaves of reddish purple Chinese cabbage have clearly different anthocyanin contents (10.170 and 32.310 g/kg DW, respectively) due to tissue differences (Rameneni et al., 2020). After exposure to low temperatures, anthocyanins are significantly promoted to accumulate in purple veins, particularly in the midribs (He Y. et al., 2020). These differences are often influenced by environmental factors due to the variations in cultivar and growing conditions.

## 3 Anthocyanin compositions and characteristics in *Brassica* crops

Anthocyanins are glycosides of hydroxyl and/or methoxy derivatives of 2-phenylbenzopyrylium or flavylium salts based on a C6-C3-C6 carbon skeleton structure (Kong et al., 2003). To date, more than twenty naturally occurring anthocyanidins have been identified, with six cyanidin, peonidin, delphinidin, petunidin, malvidin and pelargonidin—most commonly found in fruits and vegetables (Castañeda-Ovando et al., 2009). In various *Brassica* crop species, anthocyanins, including the colorful petals of the oilseed crop *B. napus*, which has high ornamental potential, have been systematically identified and analyzed. Glycosylation of anthocyanins at the 3-, 5-, and 7-positions of the anthocyanidin core has been observed in *Brassica* crops (**Table 2**). The sugars added to anthocyanins include glucose, xylose, rutinose, arabinose, galactose, sophorose, gentiotriose and glucopyranose. In *Brassica* crops, the primary anthocyanins are cyanidin 3-diglucoside-5-glucoside derivatives, which are acylated with various aromatic acids, aliphatic acids, glucosides and xylose (**Table 2**). For example, all the anthocyanins in seven red cabbage cultivars—Kosaro, Cairo, Integro, Azurro, Buscaro, Primero, and Bandolero—at two maturity stages (harvested at 13 and 21 weeks posttransplantation) consisted of nonacylated, monoacylated, and diacylated cyanidin-3-diglucoside-5-glucoside derivatives, which were acylated with sinapic, ferulic, and p-coumaric acids (Ahmadiani et al., 2014). Additionally, the sinapoyl- and feruloyl-esterified forms of cyanidin 3-diglucoside-5-glucoside are predominant in red Mizuna (Rameneni et al., 2020), whereas p-coumaryl and feruloyl esters are predominant in mustard (Xu et al., 2019). In the purple stem bark of *B. napus*, cyanidin 3-(feruloyl)diglucoside-5-(malonyl)glucoside was found to be the predominant anthocyanin. However, in the pink and red petals of rapeseed, petunidin and delphinidin derivatives are often identified as the main anthocyanin components rather than just cyanidin (Yin et al., 2019; Zeng et al., 2023), which is distinctly different from the composition found in leaves and stems.

**Table 2:** Anthocyanin compositions detected in *Brassica* crops.

Core	Types	Species/Cultivars	Reference
Cy 3-glc	Cy 3-glc	Chinese cabbage, Cauliflower, Rapeseed (petals), Red cabbage	(He et al., 2016; Scalzo et al., 2008; Yin et al., 2019; Mansour et al., 2021)
	Cy 3-(myl)glc	Chinese cabbage	(Yeo et al., 2023)
Cy 3-glc-5-glc	Cy 3-(cyl)(fyl)(syl)glc-5-glc	Purple Kohlrabi	(Park et al., 2012; Zhang et al., 2015)
	Cy 3-(fyl)glc-5-glc	Red cabbage	(Arapitsas et al., 2008; Wiczkowski et al., 2013)
	Cy 3-(p-cl)glc-5-glc	Red cabbage	(Arapitsas et al., 2008; Wiczkowski et al., 2013)
	Cy 3-(p-hl)(fyl)(myl)glc-5-(myl)glc	Bok choy sprout	(Zhang et al., 2014)
	Cy 3-(p-hl)(myl)glc-5-(myl)glc	Bok choy sprout	(Zhang et al., 2014)
	Cy 3-(p-hl)(p-hl)(myl)glc-5-(myl)glc	Bok choy sprout	(Zhang et al., 2014)
	Cy 3-(syl)glc-5-glc	Red kale sprout, Red cabbage	(Jeon et al., 2018; Arapitsas et al., 2008; Wiczkowski et al., 2013)
	Cy 3-glc-5-(myl)glc	Chinese cabbage	(He et al., 2016)
	Cy 3-glc-5-glc	Cauliflower	(Scalzo et al., 2008)
	Cy 3-(syl)glc-5-glc	Red Mizuna	(Park et al., 2020)
	Cy 3,5-diglc	Chinese cabbage, Broccoli sprout, Red cabbage, Bok choy sprout, Rapeseed (petals), Red cabbage	(He et al., 2016; Moreno et al., 2010; Arapitsas et al., 2008; Wiczkowski et al., 2013; Arapitsas et al., 2008; Wiczkowski et al., 2013; Zhang et al., 2014; Yin et al., 2019; Mansour et al., 2021)
Cy 3-diglc	Cy 3-diglc	Purple flowering stalk	(Zhang et al., 2014)
Cy 3-diglc-5-glc	Cy 3-(cyl)(syl)diglc-5-(myl)glc	Tumorous stem mustard	(Xie et al., 2014)
	Cy 3-(fyl)(fyl)diglc-5-(myl)glc	Tumorous stem mustard	(Xie et al., 2014)
	Cy 3-(fyl)(fyl)diglc-5-glc	Red cabbage, Red cabbage sprout, Tumorous stem mustard, Reddish purple Chinese cabbage	(Arapitsas et al., 2008; Wiczkowski et al., 2013) (Hrazdina et al., 1977; Xie et al., 2014), (Rameneni et al., 2020)
	Cy 3-(fyl)(syl)diglc-5-glc	Red kale sprout, Red Mizuna	(Jeon et al., 2018; Park et al., 2020)
	Cy 3-(fyl)diglc-5-glc	Purple Kohlrabi, Reddish purple Chinese cabbage, Broccoli sprout,Cauliflower, Red cabbage, Red cabbage sprout, Tumorous stem mustard, Bok choy sprout, Chinese cabbage,purpleflowering stalk,red cabbage	(Park et al., 2012; Zhang et al., 2015; Rameneni et al., 2020; Moreno et al., 2010; Scalzo et al., 2008; Arapitsas et al., 2008; Wiczkowski et al., 2013; Hrazdina et al., 1977; Xie et al., 2014; Zhang et al., 2014; He et al., 2016; Zhang et al., 2014; Pachulicz et al., 2023)
	Cy 3-(p-cl)(fyl)diglc-5-glc	Tumorous stem mustard	(Xie et al., 2014)
	Cy 3-(p-cl)(syl)diglc-5-glc	Purple Kohlrabi, Broccoli sprout,Red kale sprout, Red Mizuna, Broccoli sprout,Red kale sprout	(Park et al., 2012; Zhang et al., 2015; Moreno et al., 2010; Jeon et al., 2018; Park et al., 2020; Moreno et al., 2010),
	Cy 3-(p-cl)diglc-5-(myl)glc	Tumorous stem mustard, Red cabbage	(Xie et al., 2014; He et al., 2016)
	Cy 3-(p-cl)diglc-5-(syl)glc	Cauliflower	(Scalzo et al., 2008)
	Cy 3-(p-cl)diglc-5-glc	Tumorous stem mustard, Red Mizuna, Reddish purple Chinese cabbage, Broccoli sprout, Purple Kohlrabi,Red kale sprout, Red cabbage, Red cabbage sprout, Chinese cabbage,red cabbage, Cauliflower	(Xie et al., 2014; Park et al., 2020; Rameneni et al., 2020; Moreno et al., 2010; Park et al., 2012; Zhang et al., 2015; Jeon et al., 2018; Arapitsas et al., 2008; Wiczkowski et al., 2013; Hrazdina et al., 1977; He et al., 2016; Pachulicz et al., 2023; Pachulicz et al., 2023; Arapitsas et al., 2008; Wiczkowski et al., 2013; He et al., 2016; Scalzo et al., 2008)
	Cy 3-(p-hl)(fyl)(cyl)diglc-5-(myl)glc	Tumorous stem mustard,	(Xie et al., 2014)
	Cy 3-(p-hl)diglc-5-(myl)glc	Bok choy sprout	(Zhang et al., 2014)
	Cy 3-(syl)(fyl)diglc-5-(myl)glc	Broccoli sprout, Reddish purple Chinese cabbage, Rapeseed, Chinese cabbage	(Moreno et al., 2010; Rameneni et al., 2020; Guo et al., 2023; He et al., 2016; Moreno et al., 2010)
	Cy 3-(syl)(fyl)diglc-5-glc	Red cabbage, Reddish purple Chinese cabbage	(Arapitsas et al., 2008; Wiczkowski et al., 2013; Rameneni et al., 2020)
	Cy 3-(syl)(p-cl)diglc-5-glc	Red cabbage, Chinese cabbage	(Arapitsas et al., 2008; Wiczkowski et al., 2013)
	Cy 3-(syl)(syl)diglc-5-glc	Broccoli sprout, Purple Kohlrabi, Red cabbage, Red cabbage sprout, Red Mizuna	(Moreno et al., 2010; Park et al., 2012; Zhang et al., 2015; Arapitsas et al., 2008; Wiczkowski et al., 2013; Hrazdina et al., 1977; Park et al., 2020)
	Cy 3-(syl)diglc-5-(syl)glc	Cauliflower, Red cabbage	(Scalzo et al., 2008; Arapitsas et al., 2008; Wiczkowski et al., 2013)
	Cy 3-(syl)diglc-5-glc	Broccoli sprout, Red kale sprout, Red cabbage, Cauliflower, Red kale sprout, Red cabbage sprout, Bok choy sprout,Chinese cabbage,red cabbage, Red Mizuna, Purple Kohlrabi	(Moreno et al., 2010; Jeon et al., 2018; Arapitsas et al., 2008; Wiczkowski et al., 2013; Scalzo et al., 2008; Jeon et al., 2018; Hrazdina et al., 1977; Zhang et al., 2014; He et al., 2016; Pachulicz et al., 2023; Park et al., 2020; Park et al., 2012; Zhang et al., 2015)
	Cy 3-(cyl)(fyl)diglc-5-glc	Red cabbage, Tumorous stem mustard, Red cabbage	(Arapitsas et al., 2008; Wiczkowski et al., 2013; Xie et al., 2014; Pachulicz et al., 2023)
	Cy 3-(cyl)(p-cl)diglc-5-glc	Red kale sprout, Purple Kohlrabi, Red cabbage,red cabbage, Red Mizuna	(Jeon et al., 2018; Park et al., 2012; Zhang et al., 2015; Arapitsas et al., 2008; Wiczkowski et al., 2013; Pachulicz et al., 2023; Park et al., 2020)
	Cy 3-(cyl)(p-cl)(syl)diglc-5-glc	Purple Kohlrabi	(Zhang et al., 2015; Park et al., 2012)
	Cy 3-(cyl)(syl)diglc-5-glc	Red cabbage, Tumorous stem mustard	(Arapitsas et al., 2008; Wiczkowski et al., 2013; Xie et al., 2014)
	Cy 3-(cyl)diglc-5-glc	Red cabbage, Chinese cabbage, red cabbage	(Arapitsas et al., 2008; Wiczkowski et al., 2013; He et al., 2016; Pachulicz et al., 2023)
	Cy 3-(cyl)diglc-5-(myl)glc	Tumorous stem mustard, Reddish purple Chinese cabbage	(Xie et al., 2014; Rameneni et al., 2020)
	Cy 3-(difyl)diglc-5-glc	red cabbage	(Pachulicz et al., 2023)
	Cy 3-(disyl)diglc-5-glc	Rapeseed,red cabbage	(Guo et al., 2023; Pachulicz et al., 2023)
	Cy 3-(fyl)(cyl)diglc-5-(myl)glc	Tumorous stem mustard	(Xie et al., 2014)
	Cy 3-(fyl)(syl)diglc-5-(myl)glc	Tumorous stem mustard, Bok choy sprout	(Xie et al., 2014; Zhang et al., 2014)
	Cy 3-(fyl)(syl)diglc-5-glc	Purple Kohlrabi, Red cabbage,red cabbage	(Park et al., 2012; Zhang et al., 2015; Arapitsas et al., 2008; Wiczkowski et al., 2013; Pachulicz et al., 2023)
	Cy 3-(fyl)diglc-5-(myl)glc	Rapeseed, Tumorous stem mustard, Chinese cabbage, Reddish purple Chinese cabbage	(Guo et al., 2023; Xie et al., 2014; He et al., 2016; Rameneni et al., 2020)
	Cy 3-(fyl)diglc-5-(syl)glc	Cauliflower, Red cabbage	(Scalzo et al., 2008; Arapitsas et al., 2008; Wiczkowski et al., 2013)
	Cy 3-(gyl)(fyl)diglc-5-glc	Purple Kohlrabi	(Park et al., 2012; Zhang et al., 2015)
	Cy 3-(gyl)(syl)(fyl)diglc-5-glc	Red cabbage	(Arapitsas et al., 2008; Wiczkowski et al., 2013)
	Cy 3-(gyl)(syl)(p-cl)diglc-5-glc	Red cabbage	(Arapitsas et al., 2008; Wiczkowski et al., 2013)
	Cy 3-(gyl)(syl)(syl)diglc-5-glc	Red cabbage	(Arapitsas et al., 2008; Wiczkowski et al., 2013)
	Cy 3-(gyl)(syl)diglc-5-glc	Purple Kohlrabi	(Park et al., 2012; Zhang et al., 2015)
	Cy 3-(myl)diglc-5-glc	Red cabbage sprout	(Hrazdina et al., 1977)
	Cy 3-(oxc)(p-hl)diglc-5-glc	Chinese cabbage	(He et al., 2016)
	Cy 3-(p-cl)(p-cl)diglc-5-(myl)glc	Tumorous stem mustard	(Xie et al., 2014)
	Cy 3-(p-cl)(p-cl)diglc-5-glc	Red cabbage sprout	(Hrazdina et al., 1977)
	Cy 3-(p-cl)(syl)diglc-5-(myl)glc	Broccoli sprout, Reddish purple Chinese cabbage	(Moreno et al., 2010; Rameneni et al., 2020)
	Cy 3-(p-cl)diglc-5-(myl)glc	red cabbage	(Pachulicz et al., 2023)
	Cy 3-(p-cl)diglc-5-(succinyl)glc	Red cabbage	(Arapitsas et al., 2008; Wiczkowski et al., 2013)
	Cy 3-(p-hl)diglc-5-(oxc)glc	Red cabbage	(Arapitsas et al., 2008; Wiczkowski et al., 2013)
	Cy 3-(syl)(p-cl)diglc-5-(myl)glc	Rapeseed, Chinese cabbage	(Guo et al., 2023; He et al., 2016)
	Cy 3-(syl)(syl)diglc-5-(myl)glc	Broccoli sprout	(Moreno et al., 2010)
	Cy 3-(syl)(syl)diglc-5-glc	Red kale sprout	(Jeon et al., 2018)
	Cy 3-(syl)diglc-5-(myl)glc	Bok choy sprout, Purple flowering stalk	(Zhang et al., 2014; Zhang et al., 2014)
	Cy 3-diglc-5-(myl)glc	Bok choy sprout, Chinese cabbage, Reddish purple Chinese cabbage	(Zhang et al., 2014; He et al., 2016; Rameneni et al., 2020)
	Cy 3-diglc-5-glc	Broccoli sprout, Cauliflower, Purple Kohlrabi, Red kale sprout, Red cabbage, Red cabbage sprout, Chinese cabbage, red cabbage, Red Mizuna, Reddish purple Chinese cabbage	(Moreno et al., 2010; Scalzo et al., 2008; Park et al., 2012; Zhang et al., 2015; Jeon et al., 2018; Arapitsas et al., 2008; Wiczkowski et al., 2013; Hrazdina et al., 1977; He et al., 2016; Pachulicz et al., 2023; Park et al., 2020; Rameneni et al., 2020)
	Cy 3-(cyl)(p-cl)diglc-5-glc	Red Mizuna	(Park et al., 2020)
	Cy 3-(gpyl)(syl)diglc-5-glc	Red Mizuna	(Park et al., 2020)
Cy 3-diglc-5-diglc	Cy 3-(cyl)diglc-5-diglc	Chinese cabbage	(He et al., 2016)
	Cy 3-(fyl)(syl)diglc-5-(myl)diglc	Tumorous stem mustard	(Xie et al., 2014)
	Cy 3-(syl)(fyl)diglc-5-(myl)diglc	Chinese cabbage	(He et al., 2016)
	Cy 3-(syl)(fyl)diglc-5-diglc	Chinese cabbage	(He et al., 2016)
Cy 3-triglc-5-glc	Cy 3-(fyl)(syl)triglc-5-glc	Purple Kohlrabi, red cabbage	(Park et al., 2012; Zhang et al., 2015; Pachulicz et al., 2023)
	Cy 3-(syl)triglc-5-glc	Red cabbage, Broccoli sprout, red cabbage, red cabbage	(Arapitsas et al., 2008; Wiczkowski et al., 2013; Moreno et al., 2010; Pachulicz et al., 2023; Wiczkowski et al., 2013)
	Cy 3-(difyl)triglc-5-glc	red cabbage	(Pachulicz et al., 2023)
	Cy 3-(fyl)(fyl)triglc-5-glc	Red cabbage	(Arapitsas et al., 2008; Wiczkowski et al., 2013)
	Cy 3-(fyl)triglc-5-glc	Red cabbage,red cabbage	(Arapitsas et al., 2008; Wiczkowski et al., 2013; Pachulicz et al., 2023)
	Cy 3-(p-cl)(syl)triglc-5-glc	Purple Kohlrabi	(Park et al., 2012; Zhang et al., 2015)
	Cy 3-(p-cl)triglc-5-(myl)glc	Tumorous stem mustard	(Xie et al., 2014)
	Cy 3-(p-cl)triglc-5-glc	Red cabbage	(Arapitsas et al., 2008; Wiczkowski et al., 2013)
	Cy 3-(syl)(fyl)triglc-5-glc	Red cabbage	(Arapitsas et al., 2008; Wiczkowski et al., 2013)
	Cy 3-(syl)(p-cl)triglc-5-glc	Red cabbage	(Arapitsas et al., 2008)
Cy 3-glc-5-glc-7-glc	Cy 3-(myl)glc-5-(p-hl)glc-7-(ayl)glc	Chinese cabbage	(He et al., 2016)
	Cy 3-(myl)glc-5-(syl)glc-7-glc	Chinese cabbage	(He et al., 2016)
Cy 3-triglc-5-glc	Cy 3-(p-cl)(syl)triglc-5-glc	Red Mizuna	(Park et al., 2020)
Cy 3-diglc-5-xyo	Cy 3-diglc-5-xyo	Red cabbage	(Arapitsas et al., 2008; Wiczkowski et al., 2013)
Cy 3-glc-5-run	Cy 3-(myl)glc-5-(cyl)(fyl)run	Chinese cabbage	(He et al., 2016)
Cy 3-glc-5-run-7-diglc	Cy 3-(myl)glc-5-(p-hl)run-7-(myl)diglc	Chinese cabbage	(He et al., 2016)
Cy 3-arb-5-glc	Cy 3-(myl)(gyl)(p-hl)(p-cl)arb-5-(myl)glc	Bok choy sprout	(Zhang et al., 2014)
	Cy 3-(myl)(gyl)(p-hl)arb-5-(myl)glc	Bok choy sprout	(Zhang et al., 2014)
Cy 3-gaa	Cy 3-gaa	Rapeseed (petals)	(Yin et al., 2019)
Cy 3-glo	Cy 3-(glucopyranosyl)glo	Rapeseed (petals)	(Yin et al., 2019)
Cy 3-soh-5-glc	Cy 3-(p-cl)lsoh-5-glc	Purple flowering stalk	(Zhang et al., 2014)
Cy 3-run-5-glc	Cy 3-(cyl)(syl)run-5-glc	Bok choy sprout	(Zhang et al., 2014)
Cy 3-run-5-glc	Cy 3-run-5-glc	Purple Kohlrabi	(Park et al., 2012; Zhang et al., 2015)
Cy 3-soh-5-glc	Cy3-(syl)soh-5-glc	red cabbage	(Mansour et al., 2021)
	Cy 3-(cyl)soh-5-mylglc	Purple flowering stalk	(Zhang et al., 2014)
	Cy 3-(difyl)soh-5-mylglc	Purple flowering stalk	(Zhang et al., 2014)
	Cy 3-(fyl)(syl)soh-5-(myl)glc	Purple flowering stalk	(Zhang et al., 2014)
	Cy 3-(fyl)soh-5-(myl)glc	Purple flowering stalk	(Zhang et al., 2014)
	Cy 3-(p-cl)soh-5-(myl)glc	Purple flowering stalk, Chinese cabbage	(Zhang et al., 2014; Yeo et al., 2023)
	Cy 3-(cyl)(syl)soh-5-(myl)glc	Chinese cabbage	(Yeo et al., 2023)
	Cy 3-soh-5-glc	Chinese cabbage	(Yeo et al., 2023)
	Cy3-(fyl)soh-5-glc	red cabbage	(Mansour et al., 2021)
	Cy3-(p-cl)soh-5-glc	red cabbage	(Mansour et al., 2021)
	Cy3-soh-5-glc	red cabbage	(Mansour et al., 2021)
	Cy 3-soh-5-(myl)glc	Chinese cabbage	(Yeo et al., 2023)
Dp 3-glc	Dp 3-(cyl)glc	Chinese cabbage	(Yeo et al., 2023)
	Dp 3-glc	Chinese cabbage, Rapeseed (spetals)	(He et al., 2016; Yin et al., 2019)
	Dp 3-(fyl)glc	Chinese cabbage	(Yeo et al., 2023)
Dp 3-glc-5-glc	Dp 3,5-diglc	Chinese cabbage, Chinese cabbage	(He et al., 2016; Yeo et al., 2023)
Dp 3-glc-5-glc-7-glc	Dp 3-(fyl)glc-5-glc-7-glc	Chinese cabbage	(He et al., 2016)
	Dp 3-(syl)glc-5-glc-7-glc	Chinese cabbage	(He et al., 2016)
Dp 3-diglc	Dp 3-diglc	Purple flowering stalk	(Zhang et al., 2014)
Dp 3-diglc-5-glc	Dp 3-diglc-5-glc	Chinese cabbage	(He et al., 2016)
Pg 3-diglc-5-glc	Pg 3-(cyl)diglc-5-(myl)glc	Reddish purple Chinese cabbage	(Rameneni et al., 2020)
Pg 3-run	Pg 3-O-run	Chinese cabbage	(Yeo et al., 2023)
Po 3-diglc-5-glc	Po 3-diglc-5-glc	Chinese cabbage	(He et al., 2016)
Pt 3-glc	Pt 3-(myl)glc	Chinese cabbage	(He et al., 2016)
Pt 3-glc-5-glc	Pt 3-(myl)glc-5-glc	Chinese cabbage	(He et al., 2016)
	Pt 3,5-diglc	Bok choy sprout, Chinese cabbage	(Zhang et al., 2014; He et al., 2016)
Pt 3-diglc	Pt 3-diglc	Purple flowering stalk	(Zhang et al., 2014)
Pt 3-get	Pt 3-get	Chinese cabbage	(Yeo et al., 2023)

Cy, Cyanidin; Dp, delphinidin; Pg, pelargonidin; Po, peonidin; Pt, petunidin; glc, glucoside; run, rutinoside; arb, arabinoside; xyo, xyloside; gaa, galactoside; glo, glucopyranoside; soh, sophoroside; get, gentiotrioside; myl, malonyl; cyl, caffeoyl; fyl, feruloyl; syl, sinapoyl; p-cl, p-coumaroyl; p-hl, p-hydroxybenzoyl; gyl, glucosyl; gpyl, glycopyranosyl; ayl, acetyl; oxc, oxalic.

**Figure 2:**
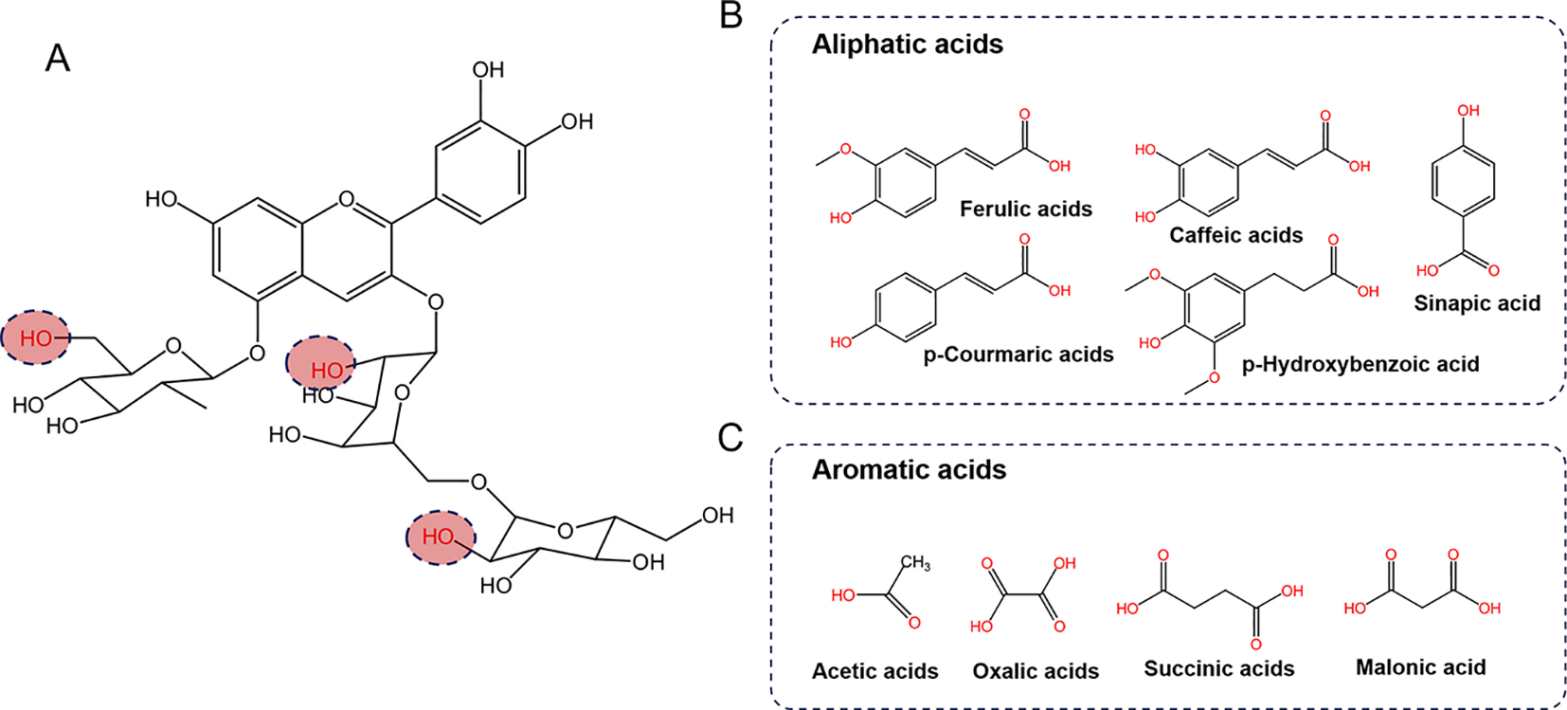
The primary anthocyanins detected in *Brassica* crops. **(A)** Chemical structures of the primary anthocyanins core, and the red dashed circles highlight the potential attachment site for acyl groups. **(B, C)** show common organic acids that acylate the sugar moiety of anthocyanins.

In general, the predominant anthocyanins are acylated cyanidin 3-glucoside/diglucoside-5-glucoside. However, anthocyanin profiles vary considerably among *Brassica* crops, particularly with respect to the types and degrees of acylation modifications present (**Table 2**; **Figure 2**). The acyl groups commonly linked to the anthocyanins in *Brassica* include aromatic acids such as p-coumaric, ferulic, sinapic, caffeic and p-hydroxybenzoic acids, as well as aliphatic acids such as oxalic, acetic, succinic, and malonic acids, in addition to glucoside, glycopyranoside and xylose (**Figure 2**).

The resilience of anthocyanins is often determined by the quantity and nature of their acyl substituents, and many studies have shown that anthocyanins with acyl groups tend to be more stable than those without such modifications (Matsufuji et al., 2007). The addition of acyl groups to anthocyanins increases their stability via both intramolecular and/or intermolecular copigmentation effects, as well as through self-association processes (Fenger et al., 2019; Cortez et al., 2017). *Brassica* anthocyanins, which feature intricate acylation patterns, display exceptional stability against thermal processing, fluctuations in pH, light exposure, and storage (Zhang et al., 2022). Prietto et al. (2017) reported that anthocyanins from red cabbage demonstrated greater stability than those from black beans when exposed to light. Leveraging this stability, they developed a pH-sensitive film utilizing extracts from red cabbage anthocyanin extracts. The extracts from red cabbage were thermostable and produced a coloring effect over a broader pH range compared to their non-acylated counterparts, such as the commercial Hibiscus-based colorant (Steingass et al., 2023). Moreover, the study of Pereira et al. (2024) suggested that the degree of acylation affects anthocyanin thermostability since extracts containing mono- and diacylated anthocyanins extracted from red cabbage are more stable than hibiscus calyxes (nonacylated) extracts are, which supports the application of polyacylated anthocyanins as natural color additives for food products.

Compared with their nonacylated forms, anthocyanins whose glycosyl moieties are acylated by hydroxycinnamic acid (HCA) residues, including caffeic, p-coumaric, ferulic, and sinapic acids, are recognized for their greater color stability (Malien-Aubert et al., 2001; Trouillas et al., 2016; Steingass et al., 2023). Purple *Brassica* crops represent promising vegetable sources for the recovery of acylated anthocyanins carrying aromatic acyl moieties such as hydroxycinnamic acids (McDougall et al., 2005). The prevailing anthocyanins in red cabbage include cyanidin-3-O-sophoroside-5-O-glucoside and its forms with one to two hydroxycinnamoyl groups attached through acetylation, which are derived primarily from p-coumaric, caffeic, ferulic, and sinapic acids (Idaka et al., 2006; Moloney et al., 2018), and red cabbage anthocyanins have been reported to be less sensitive to thermal degradation than grape, black currant, and elderberry anthocyanins are (Dyrby et al., 2001; Trouillas et al., 2016).

## 4 Biological activity and health benefits of anthocyanins from *Brassica* crops

### 4.1 Antioxidant activity of *Brassica* anthocyanins

Anthocyanins are known for their strong antioxidant activity, a property primarily attributed to their phenolic structure, which allows them to scavenge reactive oxygen species (ROS) and reduce oxidative stress. By donating hydrogen atoms or electrons, anthocyanins effectively neutralize free radicals, protecting against oxidative stress and potentially reducing the risk of aging and various diseases linked to an imbalance of free radicals and antioxidants (Lee et al., 2011). Anthocyanins from *Brassica* crops have been reported to exhibit strong antioxidant properties to protect the body from oxidative damage. *In vitro* analysis demonstrated that the extract of Hon Tsai Tai (purple *Brassica* chinensis) presented significantly greater antioxidant activity than did anthocyanin-lacking varieties such as Pak Choi and Choi Sum, as evidenced by the 1,1-diphenyl-2-picrylhydrazyl (DPPH) radical scavenging ability, reducing power, and 2’,7’-dichlorofluorescin (DCF) activity, and measurements of the intracellular superoxide dismutase activity and malondialdehyde content further confirmed its antioxidant protective effect (Chen et al., 2016). Studies on *in vivo* models have demonstrated that *Brassica* anthocyanins mitigate oxidative stress and exhibit protective effects on cellular components. For example, red cabbage anthocyanins reduce oxidative stress markers in liver mitochondria and protect plasma lipids from peroxidation in rat models (Igarashi et al., 2000; Kolodziejczyk et al., 2011).

*Brassica* anthocyanins have also been reported to prevent oxidative imbalance in brain tissue, as shown in mouse studies, where anthocyanins preserve glutathione levels in the brain under oxidative stress (Lee et al., 2002). Research also suggests that anthocyanins may modulate inflammatory responses in blood platelets, potentially through interactions with Toll-like receptor 4, which may reduce oxidative damage in inflammation-induced conditions (Saluk et al., 2015). In cellular models, such as HepG2 cells, red cabbage anthocyanins reduce H_2_O_2_-induced oxidative stress, improving cell survival and reducing apoptosis (Fang et al., 2018). Additionally, they demonstrated antioxidative effects against lipid peroxidation in rat plasma under stress, indicating broad protective effects across different biological systems (Veber et al., 2020). These findings collectively highlight the significant antioxidant potential of *Brassica* anthocyanins, which not only combat oxidative agents but also provide cellular protection, making them promising compounds for functional foods and health-promoting applications.

The effectiveness of *Brassica* anthocyanins varies across cultivars and is particularly influenced by acylation. For example, anthocyanins from the red cabbage cultivar “Langedijker Polona” presented the highest oxygen radical absorbance capacity (ORAC) value, underscoring cultivar-specific antioxidant capacities (Wiczkowski et al., 2014). Studies have shown that diacylated anthocyanins tend to be more stable and have higher antioxidant potential than monoacylated anthocyanins because of their enhanced stability and reactivity with free radicals (McDougall et al., 2007; Pereira et al., 2024). A study by Wiczkowski, Szawara-Nowak, and Topolska (Wiczkowski et al., 2013) specifically evaluated red cabbage anthocyanins and reported that compared with their nonacylated counterparts, acylated cyanidin glycosides presented greater antioxidant capacities. Additionally, the antioxidant potency of acylated anthocyanins varies depending on the type and extent of acylation (Wiczkowski et al., 2016). Among those modified with different hydroxycinnamic acids, those that are acylated with sinapic acid displayed the highest antioxidant activity (McDougall et al., 2007). Recent studies have further supported these findings, indicating that anthocyanins with multiple acyl groups, especially when bound to hydroxycinnamic acids, have not only enhanced radical-scavenging abilities but also improved stability, potentially leading to better health benefits when incorporated into diets (Zhang et al., 2021).

### 4.2 Cardiovascular protection

Anthocyanins contribute to cardiovascular health through mechanisms involving low-density lipoprotein (LDL) antioxidant and anti-inflammatory effects, as well as improvements in endothelial function. Research indicates that anthocyanins can help lower the levels of low-density lipoprotein (LDL) and very low-density lipoprotein (VLDL) under hyperlipidemic conditions and lower the risk of atherosclerosis by inhibiting lipid peroxidation and protecting vascular integrity. Al-Dosari (2014) reported that red cabbage extract decreased serum lipid levels while increasing high-density lipoprotein (HDL) levels in rats fed a cholesterol-rich diet, further supporting its hypocholesterolemic activity. Cruz et al. (2016) demonstrated that aqueous extracts of red cabbage ameliorated lipid alterations in rats induced by Triton WR-1339, leading to improved cardiovascular health. Additionally, anthocyanins from red cabbage exhibit significant hypocholesterolemic effects by influencing cholesterol metabolism (Liang et al., 2019; Zhang et al., 2022).

Moreover, studies by Sankhari et al. (2012) and Jana et al. (2017) highlighted the cardioprotective effects of anthocyanin-rich red cabbage extract, which not only preserved enzymatic and nonenzymatic antioxidants in atherogenic diet-fed rats but also provided protection against oxidative stress in myocardial infarction models. These findings underscore the potential of *Brassica* anthocyanins as functional food components for enhancing cardiovascular health. The cardioprotective effects of red cabbage anthocyanins are further supported by their ability to inhibit platelet activation, a critical factor in cardiovascular disease development. Saluk et al. (2012) reported that anthocyanins mitigate platelet hyperactivation, thereby reducing the production of reactive oxygen species (ROS), which are associated with cardiovascular risk. Overall, the consumption of *Brassica* anthocyanins appears to offer a multifaceted approach to cardiovascular protection, encompassing lipid metabolism regulation, antioxidant enhancement, and platelet activity modulation.

### 4.3 Protection against hepatic and renal injury

*Brassica* anthocyanins are valuable for combating liver and kidney damage. For example, studies have shown that red cabbage anthocyanins can alleviate liver impairment caused by high-cholesterol diets (Duchnowicz et al., 2012; Ashfaq et al., 2019). Sankhari et al. (2012) further illustrated that these anthocyanins reduced hepatic injury in rats fed an atherogenic diet by lowering triglyceride, total cholesterol, and LDL levels while increasing HDL and antioxidant enzymes such as superoxide dismutase. Turnip extracts containing anthocyanins have been shown to have hepatoprotective effects on CCl4-induced hepatotoxicity in rats by reducing the levels of serum glutamate oxaloacetate transaminase, glutamate pyruvate transaminase, and alkaline phosphatase (Sharef et al., 2019). Al-Dosari (2014) reported that red cabbage extracts effectively inhibited liver damage and demonstrated cytoprotective effects in HepG2 cells.

In addition to liver protection, red cabbage anthocyanins have also been reported to exhibit nephroprotective effects. Research by Shiyan et al. (2018) revealed that anthocyanin-rich extracts improved kidney function in rat models of gentamicin-induced nephrotoxicity. Moreover, Rezq (2018) reported that the administration of red cabbage extract to NDEA- and CCl_4_-treated rats resulted in decreased serum levels of urea nitrogen, uric acid, and creatinine, thereby improving kidney function. The protective role of these anthocyanins is further supported by findings from Sharef et al. (2019), who demonstrated that red cabbage extracts could safeguard renal tissues against gentamicin-induced nephrotoxicity. These findings suggest that incorporating anthocyanin-rich foods from *Brassica* crops into the diet may contribute positively to liver and kidney health and highlight their potential as natural therapeutic agents for managing related diseases.

### 4.4 Neuroprotective protection

Anthocyanins from *Brassica* crops also exhibit significant neuroprotective effects, primarily through the suppression of neuroinflammation and oxidative stress. Research by Lee et al. (2002) demonstrated that red cabbage extract was among the top vegetable sources that exhibited neuroprotective effects against oxidative stress in the brains of mice treated with NMDA, reinforcing the potential of these compounds in maintaining central nervous system health. Research by Heo and Lee (2006) highlighted that pretreatment with red cabbage phenolics notably inhibited amyloid-beta peptide-induced cytotoxicity in PC12 cells, indicating a protective mechanism against neurotoxicity linked to Alzheimer’s disease. Zhang et al. (2019) supported the hypothesis that red cabbage anthocyanins play a critical role in protecting neuronal cells by suppressing neuroinflammation and mitigating oxidative damage. Recently, Platosz et al. (2020) reported that the blood–cerebrospinal fluid barrier is selective for red cabbage anthocyanins and that only nonacylated derivatives are present in the cerebrospinal fluid. Furthermore, Zhang and Jing (2023) reported that extracts rich in red cabbage anthocyanins and cyanidin-3-diglucoside-5-glucoside potentially alleviated the cognitive decline associated with aging by decreasing inflammation in the nervous system, increasing antioxidant capabilities, and regulating the gut−brain axis. Collectively, these findings suggest that *Brassica* anthocyanins can serve as effective agents for neuroprotection, highlighting their importance in dietary interventions for improving cognitive health.

### 4.5 Other biological activities

**Figure 3:**
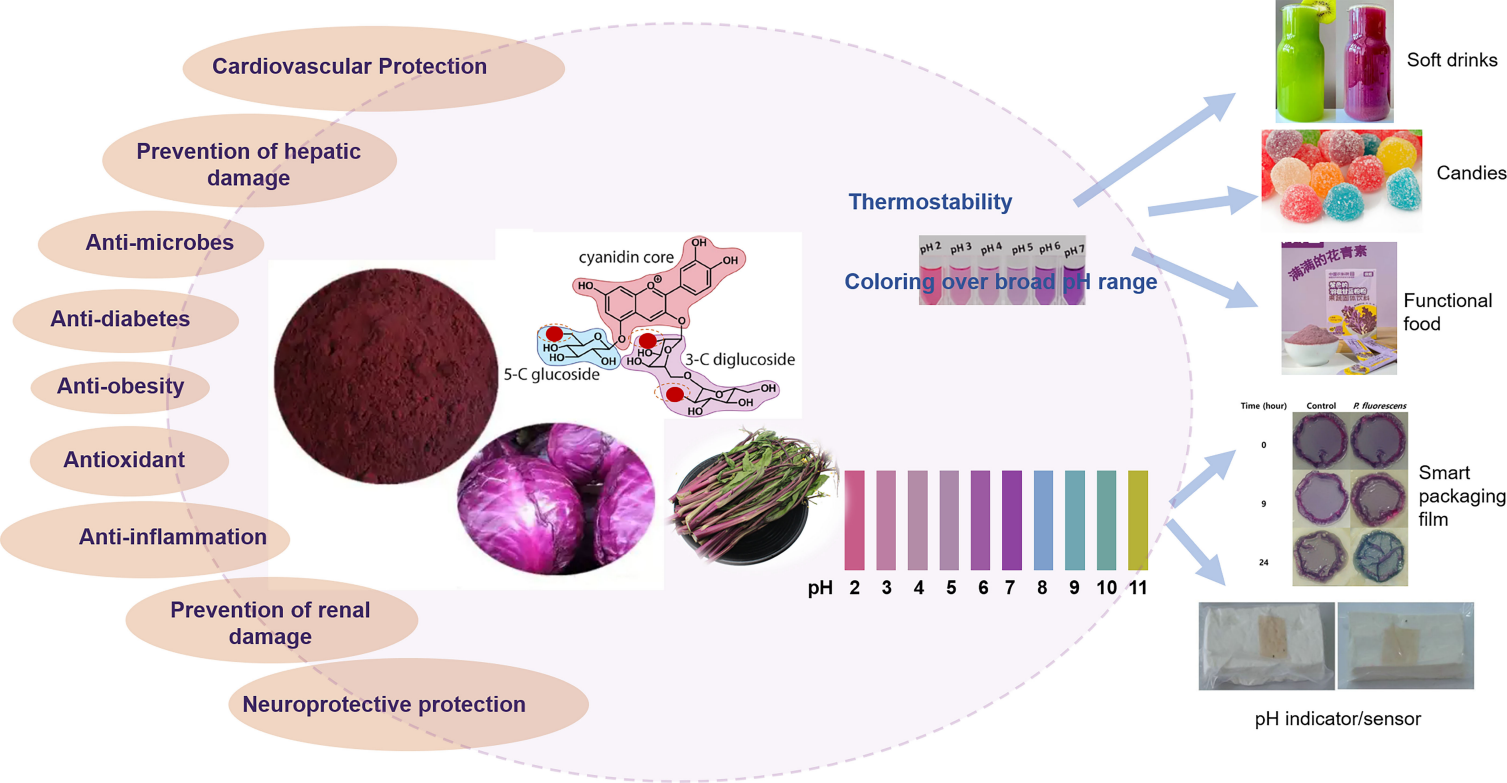
Health benefits and applications overview of anthocyanins in *Brassica* crops.

In addition, anthocyanins have been linked to various health benefits, such as anti-inflammatory, antimicrobial, antidiabetic and antiobesity effects (**Figure 3**). Zielińska et al. (2015) reported that red cabbage extracts attenuated inflammation in a mouse model of acute and chronic Crohn’s disease. Moreover, studies have demonstrated that red cabbage anthocyanins can protect against heavy metal toxicity in lymphocytes (Posmyk et al., 2009) and mitigate intestinal injuries caused by irinotecan, highlighting their potential in gastrointestinal health (Tong et al., 2017). Additionally, red cabbage extracts have shown efficacy in inhibiting pancreatic lipase, which is crucial for fat absorption, thereby assisting in weight management (Xie et al., 2018). The extracts have also been identified as effective α-glucosidase and α-amylase inhibitors, with a stronger effect against α-glucosidase, contributing to better blood glucose control and a reduced risk of diabetes-related complications (Posmyk et al., 2009). With respect to antiaging effects, research has shown that, compared with cultivars without anthocyanins, such as Pak Choi and Choi Sum, only the anthocyanin-enriched Hon Tsai Tai extract significantly prolonged the lifespan of *Caenorhabditis elegans*, resulting in an 8% increase in the mean lifespan over that of the controls (Chen et al., 2016). These diverse health benefits underscore the potential of *Brassica* anthocyanins as functional food components that may enhance overall health and prevent various diseases.

Additionally, the biological activities of anthocyanins are significantly influenced by their interactions within the colonic environment. Furthermore, bioavailability, the proportion of a nutrient that reaches systemic circulation, is a critical factor affecting the biological activity of *Brassica* anthocyanins. These aspects should be given particular attention and further investigated.

## 5 Application of *Brassica* anthocyanins

The shift toward natural colorants has been driven by consumer awareness regarding the potential adverse effects of artificial pigments on health and the environment (Rodriguez-Amaya, 2016; Francis M., 1989). Anthocyanins derived from *Brassica* crops, particularly red cabbage, have garnered attention as natural food colorants because of their vibrant hues and health benefits (Giusti and Wrolstad, 2003) (**Figure 3**). Steingass et al. (2023) recently demonstrated that, compared with commercial Hibiscus-based colorants, acylated anthocyanins from *Brassica* plants were more thermostable and produced a coloring effect over a broader pH range. Numerous studies have emphasized the suitability of *Brassica* anthocyanins as food colorants, and red cabbage-derived anthocyanins have been used in candies, soft drinks and other food products (Patras, 2019). In a recent study, Saberian et al. (2021) optimized the extraction process of red cabbage anthocyanins and explored their use as natural colorants in low-calorie gummy candies.

Owing to excellent stability and color differences across various pH values, such as pink at pH 3, violet at pH 5, and blue at pH 7 (Zhang and Jin, 2022; Dyrby et al., 2001), anthocyanins extracted from red cabbage have been widely used in the development of pH indicators for determining the freshness of food. In 2015, Silva-Pereira et al. (2015) and Pereira et al. (2015) developed pH indicators based on red cabbage anthocyanins. One of these indicators functions as a visual sign of fish decay, with superior optical and morphological characteristics. The other was designed to identify alterations in food quality by monitoring pH shifts in packaged items that had been subjected to unsuitable storage conditions. Kuswandi et al. (2020) created an edible pH sensor utilizing red cabbage anthocyanins fixed onto a bacterial cellulose membrane designed for determining pH levels in beverages and monitoring milk freshness. Moreover, Sezgin and Ocsoy (2023) developed novel colorimetric biosensors consisting of anthocyanin-rich black carrot or red cabbage extracts for rapid, sensitive, and economical detection of *Helicobacter pylori*.

Additionally, incorporating red cabbage anthocyanins in intelligent food packaging can be used to monitor the freshness of food products in real time, further contributing to food preservation (Abedi-Firoozjah et al., 2022). Nascimento Alves et al. (2020) developed biodegradable films that integrate green banana starch, gelatin, and alginate with red cabbage anthocyanins to monitor the freshness of sheep meat. These smart films are designed to detect changes in color parameters as indicators of meat quality reflected by pH changes resulting from the formation of volatile alkaline compounds during storage. Liu et al. (2021) developed colorimetric films based on polyvinyl alcohol/sodium carboxymethyl cellulose doped with red cabbage anthocyanins and demonstrated the potential of these films as indicators of freshness and intelligent packaging by monitoring the freshness of pork. Additionally, Park et al. (2022) investigated the antimicrobial activity and indicator properties of edible chitosan-based films prepared with red cabbage anthocyanins (as spoilage indicators) and clove bud oil (as antimicrobial agents), and the results indicated that the color change of the films reflected the growth process of the fish-spoiling bacteria.

Nevertheless, like anthocyanins from other species, the utilization of *Brassica* anthocyanins in the food and medical sectors has been significantly hindered by their inadequate stability and bioavailability (Chen Y. et al., 2023; González-Barrio et al., 2010). To enhance the stability of ACNs, a variety of approaches have been employed. These include encapsulation techniques such as spray-drying, freeze-drying, and the hard-panned coating method, as well as co-pigmentation and innovative enzymatic methods (Cortez et al., 2017), with nanoencapsulation also emerging as a promising strategy (Liang et al., 2024). Wagh et al. (2023) fabricated a new generation of carbon dot-based active and intelligent packaging films with antibacterial, UV blocking and real-time sensing potential via *B. oleracea*(BO) extract. The packaging trials demonstrated that the developed film operated effectively and without causing damage, enabling real-time monitoring of the freshness of ground pork, fish, and shrimp. The film indicated freshness through a visible transition from red to colorless/yellow, indicating its potential as a multifaceted packaging solution. This material can signal quality deterioration and prolong the shelf-life of perishable packaged goods.

Overall, the versatility of *Brassica* anthocyanins as natural colorants, coupled with their application in food monitoring and smart packaging solutions, position them as valuable components in the food industry, promoting health, safety, and sustainability.

## 6 Conclusion

This review underscores the multifaceted significance of anthocyanins in *Brassica* crops, which are not only rich in these pigments but also exhibit a broad spectrum of biological activities and health benefits. From their antioxidant and anti-inflammatory properties to their potential in cardiovascular health and neuroprotection, anthocyanins have emerged as vital components in functional foods. Moreover, their application extends beyond nutrition, serving as natural colorants and integral to smart packaging solutions for real-time food freshness monitoring. The stability and pH-responsive color changes of these anthocyanins make them ideal for developing indicators in food technology, emphasizing their role in enhancing food safety and quality. This comprehensive overview highlights the potential of *Brassica* anthocyanins as key players in the food industry, contributing to health, sustainability, and technological advancement.
